# An Overview on Acute Malnutrition and Food Insecurity among Children during the Conflict in Yemen

**DOI:** 10.3390/children6060077

**Published:** 2019-06-05

**Authors:** Fekri Dureab, Eshraq Al-Falahi, Osan Ismail, Lina Al-Marhali, Ayoub Al Jawaldeh, Nazmun Nahar Nuri, Elvis Safary, Albrecht Jahn

**Affiliations:** 1 Modern Social Association (MSA), Aden, Yemen; almarhalilina@gmail.com; 2 Heidelberg Institute of Global Health, Hospital University, Heidelberg 69115, Germany; nuri.nazmun-nahar@uni-heidelberg.de (N.N.N.); elvisafary@gmail.com (E.S.); Albrecht.Jahn@uni-heidelberg.de (A.J.); 3 World Health Organization (WHO), P.O. Box 543, Sana’a, Yemen; alfalahie@who.int (E.A.-F.); ismailo@who.int (O.I.); 4 World Health Organization, EMRO, P.O. Box 7608, Nasr City, Cairo 11371, Egypt; aljawaldeha@who.int

**Keywords:** malnutrition, food insecurity, infection, Yemen

## Abstract

Background: This study aims to describe malnutrition among children under five and to describe the food insecurity status during the current conflict in Yemen. Methods: Data were obtained from a Yemeni nutrition surveillance program (pilot phase) targeting 4142 households with 5276 children under five from two governorates (Ibb and Sana’a). Results: Global acute malnutrition was found in 13.3% of overall screened children, while 4.9% had severe acute malnutrition (SAM) and 8.4% had moderate acute malnutrition. One-fifth of the children under six months of age were acutely malnourished, followed by children under two years at 18.5% based on weight-for-height z scores. Significant associations between malnutrition and other diseases included suspected measles at three times higher rates (4.5%, *p* < 0.00) among SAM cases than other children. Diarrhea, fever, and cough were significantly higher among the SAM group (*p* < 0.05). Most households depended on market food purchases in the month preceding this survey (84.7%). Household coping mechanisms to secure daily meals included borrowing food to survive, changing types and quality of food, and decreasing the number of meals per day; some families sent their children to live with relatives. Conclusion: Malnutrition is a serious public health problem. The humanitarian community needs to adopt alternative strategies to improve food security and the nutrition status in Yemen.

## 1. Introduction

Yemen is the poorest country in the Middle East and North Africa, and since March 2015 it has been facing a significant humanitarian and security crisis. The ongoing intensified war and disrupted social services have had a clear impact on the general health and nutrition status of the children and entire population [1]. The food distribution mechanism in the country has been severely disturbed. Yemen is highly dependent on imported food, and the current armed conflict has blocked food transportation, which has caused a food crisis. The decrease in food availability has led to an increase in food costs, although the conflict has devastated purchasing power as a result of increased unemployment and loss of family incomes [2,3].

Yemen is facing the largest food security emergency in the world because of a severe decline in agriculture production, which affects approximately 60% of Yemenis who depend on agriculture for their livelihoods [4]. According to the Integrated Food Security Phase Classification (IPC), 13 of 22 governorates are experiencing catastrophic conditions (IPC phase 5), 17% of the population (5 million) are classified in an emergency phase (IPC phase 4), and 36% (approximately 10.8 million) are in a crisis phase (IPC phase 3) [5]. Yemen has one of the highest rates of chronic malnutrition, with estimates of stunting in 47% of children under five and wasting in 15% [6].

The United Nations (UN) has estimated that 22.2 million people in Yemen need some kind of humanitarian assistance. The UN report also notes that approximately 17.8 million are food insecure and 16.4 million lack adequate healthcare. A total of 1.8 million children are acutely malnourished, and 462,000 children suffer from severe acute malnutrition (SAM) [7]. Conflict is driving people from their homes at alarming rates since an estimated 2 million people are currently displaced within Yemen [8]. Since there are no camps for internally displaced persons (IDPs), displacement has led to a dispersed population that is often difficult to identify or assess for vulnerability or specific needs. Prior to the conflict’s escalation in March 2015, significant humanitarian needs already existed across Yemen [9]. In Hodeida, approximately 100,000 children under the age of five are at risk for SAM compared to 23,000 before 2015. In Aden, the figure more than doubled and is currently 7700 compared to 3000 before 2015 [10].

The most common diseases associated with acute malnutrition are respiratory infections. Pneumonia is common in malnourished children and leads to fatal complications [11]. Pneumonia and diarrheal diseases account for approximately 27% of the mortality of children under five in Yemen [12]. Globally, malnutrition is responsible for nearly half (45%) of all deaths of children under the age of five [13]. Together with poor diet, malnutrition is the number one driver of the global burden of disease. According to WHO, 5.9 million children worldwide died under the age of five in 2015, although the deaths were due to preventable and curable conditions if the children had access to simple, affordable interventions [13].

This study aims to determine the prevalence and factors associated with acute malnutrition among children under five years in the communities of Ibb and Sana’a governorates during the current conflict in Yemen.

## 2. Materials and Methods

This study analyzed data that were obtained from the Yemeni nutrition surveillance program. The nutrition surveillance system is an active, population-based surveillance, started as a pilot in two governorates (Ibb and Sana’a). The survey was conducted by 88 trained field investigators, distributed over 18 districts in 234 clusters (villages). The total study population included 4142 households and a total of 5276 children under five years old. The survey was conducted by health staff from health facilities in the same districts. Several variables in nutrition and health were used in this study, Table 1 shows definitions of some variables.

Sampling was stratified by administrative units and samples among clusters of geographic units (villages, urban segments, or statistical enumeration areas). The surveillance design had three stages of sampling (cluster–household–respondent). Probability proportional to size (PPS) sampling was the primary methodology for selecting clusters, while random sampling was used at the household level. 

Data were analyzed using Emergency Nutrition Assessment (ENA) software and SPSS version 25 (IBM Corp., Armonk, NY, USA). Outcomes were compared between the two governorates. Chi-square tests were applied to examine the association between malnutrition and associated health factors. Furthermore, logistic regression was applied to examine the effects of malnutrition on the children’s general health status. The result of the regression analysis was presented by odds ratios (ORs) with 95% confidence intervals (CIs).

### Ethical Consideration

An official approval was obtained from the Ministry of Public Health and World Health Organization in Yemen to use these data for further analysis.

## 3. Results

The total study population of children was 5276. Approximately 52.6% of the children were male. One-third of the children were under two years old; mean age was 31.25 months (SD ± 16.56), mean height was 82.61 cm (SD ± 12.90), and mean weight was 10.63 kg (SD ± 3.10) (see Table 2).

The total number of acutely malnourished children according to weight-for-height z scores (WHZ) was 706 of the total 5276. Global acute malnutrition (GAM) was found in 13.3% of all screened cases, while 4.9% had SAM and 8.4% had moderate acute malnutrition.

Children under six months are the most affected by acute malnutrition. Table 3 shows that 20% of the children under six months in Ibb and Sana’a were acutely malnourished, followed by children under two years of age (18.5%) based on WHZ.

There are many factors that affect the nutritional status of children in Yemen, and Table 4 provides examples. Approximately 43% of the children under six months were still exclusively breastfed during the survey in both governorates (55% in Ibb and 33% in Sana’a). The majority of children had been vaccinated completely or partially in Ibb (93.9%) and Sana’a (92%), although 1.7% of children in Ibb and 1.4% in Sana’a had a history of suspected measles in the week prior to the survey. Diarrhea, fever, and cough were common in both governorates: 27.4%, 30.7%, and 29.3%, respectively, in Ibb and 25.1%, 31.3%, and 33.2%, respectively, in Sana’a.

Table 5 shows associations of the most common health problems and malnutrition in children under five years old. There were significant associations between malnutrition and other diseases. For instance, suspected measles was three times higher (4.5%, *p* < 0.00) among SAM cases than other children. Similarly, diarrhea (38%), fever (37.7%), and cough (38.6%) were significantly higher among children with SAM than other children (*p* < 0.05).

### Food Security at the Household Level

The mean number of household members was 7.5 persons (SD ± 3.922), and the mean number of children under five years old was 1.34 children (SD ± 1.166). The mean daily meals for children under two years was 1.37 meals (SD ± 1.876) and was 2.09 meals (SD + 1.843) for those 24–59 months.

Table 6 shows that families had various sources of food, and most depended on purchasing food from the market in the month preceding this survey (84.7%). Families in the Sana’a governorate depended more on food purchasing than those in Ibb. Agriculture was the second source of food overall (30.5%). Receiving food from humanitarian assistance was the third most common source overall (10.9%), comprising 16.6% in Sana’a governorate and 5.7% in Ibb.

**Figure 1:**
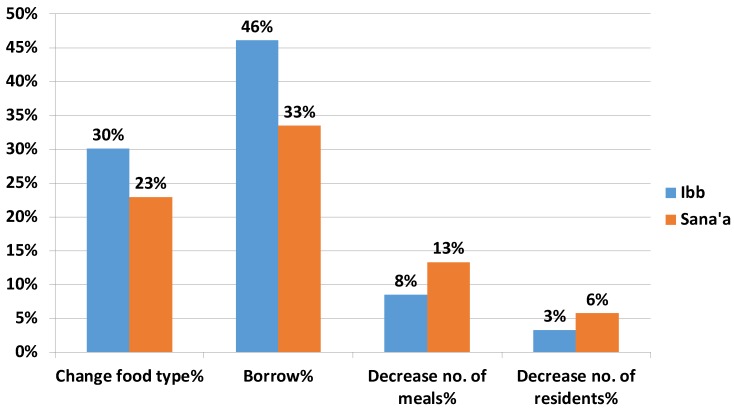
Distribution of coping mechanisms for food insecurity among families in the target communities of Ibb and Sana’a by governorate (multiple answers possible).

Figure 1 shows that families in the targeted communities used various coping mechanisms to secure daily meals. Approximately 40% of the families borrowed food to survive (46% in Ibb and 33.4% in Sana’a). One-quarter of the families changed the type and quality of their food to mitigate household food shortages (30% in Ibb and 23% in Sana’a). Decreasing the number of meals was also an option for 10.8% of poor families overall, comprising 8.5% in Ibb and 13.3% in Sana’a. Other families sent their children to live with relatives to reduce the number of people in the household: 3.3% in Ibb and 5.7% in Sana’a.

## 4. Discussion

GAM for both Ibb and Sana’a governorates was 13.3% (WHZ). This high prevalence of acute malnutrition is classified as a critical phase, although it does not exceed the emergency threshold 15% [14]. The national rate of GAM for Yemen was 16% in the last demographic health survey (DHS) in 2013 [6]. However, the Nutrition Cluster report (SMART) in April 2017 showed that the GAM rates in both governorates were classified as poor (between 5% to 9.99%) [15]. The difference in GAM rates between this survey and SMART 2017 could be due to seasonal differences that made comparisons invalid or the seasonal impact of nutrition interventions in both governorates.

The SMART and Yemen Emergency Food Security and Nutrition Assessment (EFSNA) surveys in Ibb and Sana’a did not indicate a major deterioration in the prevalence of GAM compared to levels before March 2015 (preconflict) [16,17,18]. However, food security surveys indicated an obvious deterioration in the outcomes in Ibb and Sana’a governorates [19]. In 2017, IPC indicated that the deterioration had increased by 20% compared to the results of IPC 2016 [5]. Despite the ongoing conflict and deterioration of the economic situation in Yemen, IPC 2018 showed an improvement in food security by 7% compared to 2017. On the negative side, the same report showed that 0.2% of the population (63,500) was in a catastrophic phase of food insecurity (IPC phase 5) [20], thus an impact study was recommended after more than three years of humanitarian work in Yemen.

The severity of acute malnutrition decreases with the children’s increasing age. The prevalence of acute malnutrition among children under two years was much higher than children 24–59 months. This finding is in line with studies in India that also report a high prevalence of malnutrition among children under two years old [21,22]. This clearly indicates a relationship between malnutrition and the practice of infant and young child feeding in these communities; this age is a period for physical growth and an increase in metabolic demands. Therefore, more energy and nutrients, more than what is provided by breast milk and complementary feeding, are needed [23]. The practice of breast feeding is low in these communities—only 24% of the children under two years were still breastfed during the study period in Yemen. On the other hand, SMART 2017 in Ibb found that 63% of the children were still breastfed, and similar results were found in Sana’a (67%) in the SMART 2016 report. Several studies showed that improper feeding practices of young children were associated with malnutrition in poor communities [22,24]. This Yemeni study revealed that the mean number of meals taken by the children per day was very low: 1.37 meals for children between 6–23 months and 2.09 meals for children of age 24–59 months.

Morbidity is more common among malnourished children. In this Yemeni study, 31% of the children under five had complained of fever within the week preceding the survey in both governorates. This finding was close to the level of fever prevalence reported in the 2013 DHS (32%). The prevalence of diarrhea was 26.5% among children within the week preceding the survey, which was less than the national prevalence in the DHS 2013 report (31%). A history of cough and acute respiratory infection (ARI) among children under five within the week preceding the survey was 31% in both governorates, while in DHS 2013 it had been 12%; however, rates of ARI were high in SMART surveys of Ibb 2017 and Sana’a 2016 (42% and 50%, respectively).

There were significant associations between SAM and other diseases. This study revealed that suspected measles was three time higher among children with SAM than others (4.5%; *p* < 0.05). Many studies have shown that measles may cause malnutrition, and the nutritional status of children may also determine the severity of measles [25]. Diarrhea, fever, and cough rates were significantly higher among children with SAM in this study (*p* < 0.05). The risk of illness upsurged significantly with an increasing severity of malnutrition in children [26].

The reported vaccination coverage in this study was high (>90%); however, the national immunization coverage reported by national authorities was estimated at 83% for DPT3 (Diphtheria, Tetanus and Pertussis) in 2017, and WHO/UNICEF estimated it at 68% [27]. Immunization coverage is severely affected by the ongoing conflict. Immunization services are available in only 35% of functional health facilities in the county [28].

The deterioration of food security in Yemen is expected to continue as long as the conflict is ongoing, thus affecting household livelihoods and the general marketing situation. Food prices have sharply increased in local markets, which deprives the majority of this survey population who depend on the local market to get their daily food. In addition to high prices, other challenges include a reduction in family income from farming, fishing, or governmental salaries [29].

Approximately 30% of the study population depended on agriculture as their food source, particularly in Ibb. Approximately 11% depended on humanitarian assistance overall in Yemen, comprising 17% in the Sana’a governorate and 6% in the Ibb governorate. However, in June 2016, the report of the food security cluster revealed that 29% of the study population in Ibb was reached by humanitarian food assistance, and it was a much higher number (56%) in the Sana’a governorate [30]. Currently, food insecurity is a major public health problem in Yemen, and it is having significant social implications as households have tried multiple coping mechanisms to address their hunger. Borrowing food was observed to be the first step to mitigate the adverse effect of food insecurity at the household level in Yemen. In Bangladesh, the majority of poor households primarily chose borrowing money to buy food and compromised on the quality and quantity of food [31]. Unfortunately, households in Yemen also chose among these tough options, including sending their children to live with other families.

This study has some limitations. There were (and still are) challenges to obtain reliable data from mothers or relatives of children, for example, with regard to history of measles and fever during the last ten days prior to this survey. Health staff depended on relatives to recall information regarding the vaccination status of children, which had a high probability of recall bias.

## 5. Conclusions

Malnutrition among children is a serious public health problem in Yemen, and the severity of acute malnutrition increases among children under two years. The majority of families in Yemen depend on food purchasing from the market, which is itself a disaster because of economic deterioration and lack of salaries. The question that needs further investigation is: Does humanitarian assistance have an impact in Yemen, and what is the extent of this impact? We recommend that the humanitarian community adopt alternative strategies to improve food security and nutrition status in Yemen. Income-generating projects to improve the resilience of the vulnerable Yemeni population is one of the recommendations.

## Figures and Tables

**Table 1:** Definitions.

Variable	Definition
Exclusively breastfed	The infant under six months was only breastfed. No other liquids (even water) or solids were given, with the exception of medicine.
Breastfeeding during the survey	The child was still breastfed up to the age of two
Suspected measles	The child was suffering from fever, rash, and cough and/or conjunctivitis, and they were diagnosed clinically as suspected measles by medical staff in a health facility.
Diarrheal	Passage of three or more loose stools per day
Fever	Temperature identified by mothers above the normal (subjective)
Vaccination status	If child had all vaccinations or some of them (BCG for TB, OPV for Polio, Pentavalent, Rotavirus C, and MCV for Measles)

**Table 2:** Demographic characteristics of children under five in Ibb and Sana’a governorates.

Characteristics		Children	
		Number	Percentage
Governorate	Sana’a	2368	45%
	Ibb	2908	55%
Sex	Male	2777	52.6%
	Female	2499	47.4%
Age group	>6 months	270	5.1%
	6–23 months	1522	28.8%
	24–59 months	3484	66%

**Table 3:** Distribution of the nutritional status of children under five years of age in Ibb and Sana’a governorates by MUAC * and WHZ ** according to age group.

Classification.	Moderate Acute Malnutrition		Severe Acute Malnutrition		Global Acute Malnutrition	
	WHZ	MUAC	WHZ	MUAC	WHZ	MUAC
<6 months	23 (8.5%)		31 (11.5%)		54 (20%)	
6–23 months	175 (11.5%)	267 (17.5%)	106 (7%)	189 (12.4%)	281 (18.5%)	456 (29.9%)
24–59 months	247 (7%)	286 (8.8%)	120 (3.4%)	122 (3.5%)	367 (10.5%)	408 (11.6%)
Total	445 (8.4%)	600 (11.3%)	257 (4.9%)	337 (6.3%)	702 (13.3%)	937 (17.6%)

* MUAC, mean upper arm circumference; ** WHZ, weight-for-height z score.

**Table 4:** Factors affecting the nutritional status of the targeted children under five years of age in Ibb and Sana’a governorates.

Health Factors		Ibb		Sana’a	
		N	%	N	%
Exclusively breastfed children under six months	Yes	61.0	55%	44.0	33%
	No	50.0	45%	89.0	67%
Breastfeeding during the survey time	Yes	704.0	24.2%	559.0	23.6%
	No	2214.0	74.8%	1814.0	76.4%
Suspected measles history in the week prior the survey	Yes	49.0	2%	33.0	1.4%
	No	2871.0	98%	2340.0	98.6%
Diarrheal history in the week prior the survey	Yes	800.0	31.8%	596.0	25%
	No	2121.0	68.2%	1777.0	75%
Fever history in the week prior the survey	Yes	897.0	35.3%	742.0	31.3%
	No	1644.0	64.7%	16310	68.7%
Cough history in the week prior the survey	Yes	856.0	33.5%	788.0.	33.2%
	No	1703.0	66.5%	1585.0	66.8%
Vaccination status	Yes	2449.0	93.9%	2029.0	92%
	No	82.0	3.1%	102.0	4.6%
	Unknown	77.0	3.0%	76.0	3.4%

**Table 5:** Associations between health problems and the nutritional status of children under five years of age in the targeted areas of Ibb and Sana’a governorates.

Health Factors—History of Disease in the Week Prior to Survey		Adequate Nutritional Status		Moderate Acute Malnutrition		Severe Acute Malnutrition		*p*-Value Chi-Square
		N	%	N	%	N	%	
suspected measles	Yes	60	1.4%	7	1.2%	15	4.5%	*p* < 0.000
	No	4305	98.6%	584	98.8%	322	95.5	
Diarrhea	Yes	1105	25.3%	162	27.4%	128	38%	*p* < 0.000
	No	3260	74.7%	429	72.6%	209	62%	
Fever	Yes	1337	30.6%	180	30.5%	127	37.7%	*p* < 0.025
	No	3028	69.4%	411	69.5%	210	62.3%	
Cough	Yes	1320	30.2%	189	32.0%	130	38.6	*p* < 0.005
	No	3045	69.8	402	68.0%	207	61.4%	

**Table 6:** Distribution of food sources among families in the target communities in Ibb and Sana’a by governorate (multiple answers possible).

Source of Family Food	Ibb N (%)	Sana’a N (%)	Total N (%)
Agriculture	715 (32.1%)	582 (28.9%)	1297 (30.5%)
Market	1848 (83%)	1743 (86.6%)	3591 (84.7%)
Humanitarian Assistance	127 (5.7%)	334 (16.6%)	461 (10.9%)
